# Liquid Metal Gallium Promotes the Activity and Stability of the Cu-ZnO Catalyst for CO_2_ Hydrogenation to Methanol

**DOI:** 10.3390/molecules30204033

**Published:** 2025-10-10

**Authors:** Yu Zhang, Yuanshuang Zheng, Xiulin Wang, Suofu Nie, Wenqian Zhang, Lun He, Bang Gu

**Affiliations:** 1 CNOOC Key Laboratory of Liquefied Natural Gas and Low-Carbon Technology, Beijing 100028, China; 2 CNOOC Gas & Power Group Co., Ltd., Beijing 100028, China; 3 School of Chemical Science and Technology, Yunnan University, 2 North Cuihu Road, Kunming 650091, China

**Keywords:** CO_2_ hydrogenation, methanol, liquid metal gallium, physical mixing, long-term stability

## Abstract

CO_2_ hydrogenation to methanol has attracted considerable attention as a promising catalytic route for both reducing CO_2_ emissions and producing valuable chemical intermediates. Among various catalysts, Cu–ZnO-based systems are the most widely studied; however, their performance remains constrained by limited methanol selectivity and stability, highlighting the need for improved catalytic strategies. In this work, liquid metal gallium (Ga) was incorporated into Cu–ZnO catalysts as an additive for CO_2_ hydrogenation to methanol. Owing to its high dispersibility and fluidity, Ga helps maintain long-term catalyst stability. We investigated different introduction methods for Ga promoters and found that the physical mixing approach generated the strongest alkaline sites, thereby enhancing CO_2_ activation and increasing the CO_2_ conversion to methanol. Moreover, this catalyst effectively suppressed carbon deposition, further improving its stability. These findings offer new insights into the use of liquid metal Ga in CO_2_ hydrogenation and provide fresh perspectives for the rational design of efficient methanol synthesis catalysts.

## 1. Introduction

The large-scale combustion of fossil fuels has significantly accelerated socioeconomic development. However, it has also led to serious global climate challenges, including global warming, ocean acidification, the intensification of the greenhouse effect, and water pollution [1,2,3]. As a result, the efficient utilization of CO_2_ has emerged as a major research focus within both the scientific and industrial sectors [4,5]. CO_2_ is increasingly recognized not merely as a greenhouse gas but rather as a potential resource. The utilization of CO_2_ as a key C1 molecular platform for catalytic conversion offers a promising pathway not only to mitigate greenhouse gas accumulation and reduce net carbon emissions, but also to generate valuable chemicals critical to modern society [6]. This approach facilitates the production of essential C1 compounds such as methanol, methane, synthesis gas, and formic acid, as well as C_2+_ products, thereby contributing to energy security and sustainable resource utilization [7,8].

Methanol serves as an efficient liquid hydrogen carrier and constitutes one of the most fundamental feedstocks in modern chemical engineering. It holds a strategically essential role within the global energy transition and the broader chemical value chain [9,10]. The catalytic hydrogenation of CO_2_ to methanol has been extensively investigated, with Cu-ZnO-based catalysts representing the most widely studied system [11,12]. However, the CO_2_ conversion and methanol selectivity of Cu-ZnO catalysts remain unsatisfactory. To address these limitations, numerous studies have explored the incorporation of metal promoters, among which Ga has proven to be highly effective. For example, Tsang et al. employed a coprecipitation method to incorporate Ga^3+^ into Cu-ZnO catalysts. They proposed that the promoting effect of Ga^3+^ doping originates from the formation of the ZnGa_2_O_4_ spinel structure. Furthermore, by increasing the ZnO content within the catalyst, both the CO_2_ conversion and methanol selectivity could be significantly enhanced [13]. Gu et al. synthesized a ternary CuZnGa catalyst via a one-pot method and confirmed that the introduction of Ga leads to the formation of a ZnGa_2_O_4_ spinel structure. This structure was found to effectively enhance CO_2_ activation while suppressing the reverse water–gas shift (RWGS) reaction, thereby improving methanol selectivity [14]. Taken together, these studies highlight the notable promotional effect of Ga on CO_2_ hydrogenation to methanol. However, most existing research focuses on Ga^3+^ ion or spinel structures. In contrast, liquid metal Ga has recently attracted increasing attention due to its fluidity, low melting point, tunable surface properties, and exceptional anti-coking performance, which collectively contribute to long-term catalyst stability [15,16]. Nevertheless, no studies have yet explored the use of liquid metal Ga to improve the performance of Cu-ZnO catalysts, and its underlying mechanism remains to be systematically investigated.

In this study, the influence of liquid metal Ga over Cu-ZnO catalysts for CO_2_ hydrogenation to methanol was systematically investigated, leveraging the unique properties of liquid metal Ga such as its fluidity and surface tunability and cleaning. Different introduction methods of gallium, namely impregnation (CuZnGa-i) and physical mixing (CuZnGa-p), on the catalytic performance of Cu/ZnO catalyst were also examined. Characterization results indicate that liquid gallium exhibits carbon deposition suppression capabilities, thereby enhancing catalytic stability. Among the preparation methods evaluated, the catalyst with liquid metal Ga introduced by physical mixing achieved the highest CO_2_ conversion, the greatest methanol selectivity, and superior long-term stability. This work provides valuable insights into the application of liquid gallium as a catalytic component for enhancing methanol production via CO_2_ hydrogenation.

## 2. Results

### 2.1. Structures of Catalysts

**Figure 1:**
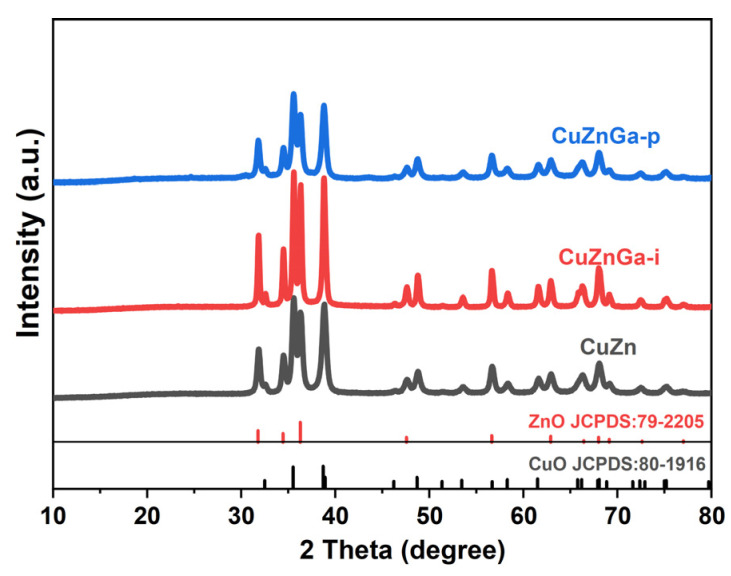
XRD diffraction patterns of the fresh samples.

The crystalline phases of the catalyst materials were examined by XRD, and the resulting patterns are presented in Figure 1. The XRD pattern of the CuZn catalyst shows diffraction peaks corresponding exclusively to ZnO (JCPDS 79-2205) and CuO (JCPDS 80-1916). No additional phases were detected, which is consistent with the expected phase composition. The XRD pattern of the CuZnGa-i and CuZnGa-p catalysts closely resembles that of the binary CuZn catalyst. And no diffraction peaks attributable to Ga or its oxides were detected in the CuZnGa-i and CuZnGa-p catalysts. This absence suggests that the Ga species are highly dispersed, likely existing as amorphous metals or integrated into the lattice of the support or other components [17]. The simultaneous presence of phases CuO and ZnO confirms a composite crystal structure. Owing to the high molar content of Cu and Zn, the diffraction peaks from the CuO and ZnO phases are the most intense features in the patterns of all three catalysts. Furthermore, the introduction of liquid metal Ga by the physical mixing method appears to have little effect on the CuZn crystal structure, whereas the impregnated CuZnGa-i catalysts enhanced the overall crystallinity of the catalyst.

**Figure 2:**
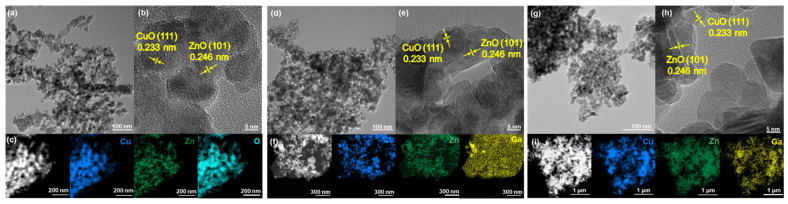
(**a**–**i**) HRTEM images and STEM-EDS elemental mapping of used CuZn (**a**–**c**), CuZnGa-i (**d**–**f**), and CuZnGa-p (**g**–**i**).

Furthermore, to comprehensively investigate the morphology, composition, and crystal structure of the catalysts, TEM and HRTEM characterization were performed on all three samples. Observation of Figure 2a,b, Figure 2d,e, and Figure 2g,h indicates that all three catalysts feature well-defined particle structures, demonstrating morphological similarity to conventional Cu-based catalysts. HRTEM analysis reveals discernible lattice fringes with interplanar spacings of 0.233 nm and 0.246 nm across all three catalysts, which are assigned to the (111) plane of CuO and the (101) plane of ZnO, respectively. Our analysis failed to identify any crystal planes associated with Ga, which aligns with the XRD data. EDS mapping was also performed on the post-reaction catalysts (Figure 2c,f,i). The uniform distribution of all elements confirms the high structural stability of the catalysts, whereas the CuZnGa-i catalysts exhibit Ga aggregation after the reaction. In contrast, the Ga species in the CuZnGa-p catalyst show a remarkably uniform and extensive distribution without significant agglomeration. This behavior is attributed to the high flowability of liquid Ga, which enhances the catalyst’s stability during the reaction. In addition, EDS analysis confirmed that the elemental composition of the catalyst matched the expected stoichiometric ratio and almost no change before and after the reaction (Table 1).

### 2.2. Chemisorptive and Reductive–Hydrogenative Properties of Catalysts

**Figure 3:**
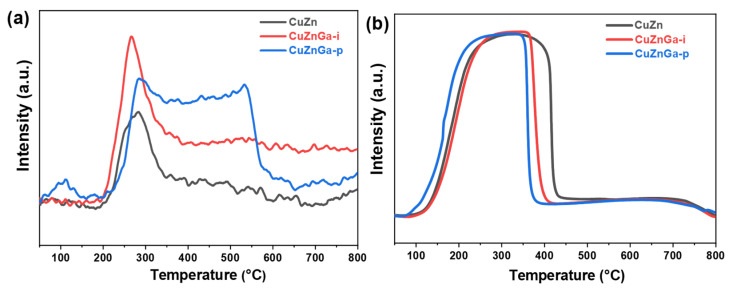
(**a**) CO_2_-TPD profiles and (**b**) H_2_-TPR profiles of fresh CuZn, CuZnGa-i, and CuZnGa-p catalysts.

As shown in Figure 3a,b, we conducted investigations to evaluate the chemical adsorption and reduction behavior of the catalyst. CO_2_ adsorption and desorption are crucial processes in CO_2_ hydrogenation to methanol. Characterization demonstrates that only the CuZnGa-p catalyst exhibits a low-temperature desorption peak near 100 °C, indicative of weak physical adsorption on its surface. Furthermore, the desorption peaks observed in the mid-temperature range (200–600 °C) are predominantly attributed to the presence of alkaline oxygen vacancies, which are primarily responsible for CO_2_ activation [18,19]. The broad peak here may consist of a primary desorption peak at 200–400 °C and a secondary desorption peak above 400 °C. The primary desorption peak in the mid-temperature range primarily serves to activate CO_2_, while the secondary desorption peak in the high-temperature range promotes the occurrence of side reactions. All three catalysts exhibit distinct CO_2_ desorption peaks. Notably, the incorporation of liquid gallium significantly enhances the total desorption area, which is most pronounced for the CuZnGa-p catalyst. The largest desorption area correlates with its superior CO_2_ activation capacity. H_2_-TPR profiles of the three catalysts exhibit similar reduction curves with largely comparable peak areas. The incorporation of liquid gallium is observed to slightly lower the reduction temperature of Cu-ZnO-based catalysts [20].

**Figure 4:**
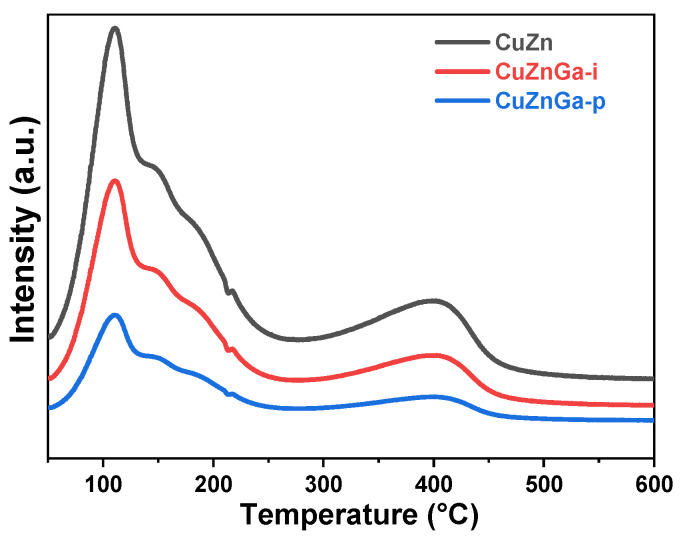
H_2_-TPH of used CuZn, CuZnGa-i, and CuZnGa-p catalysts.

Carbon deposition is recognized as a primary mechanism of catalytic deactivation. Excessive carbon accumulation can block key active sites on the catalyst surface, resulting in a significant decline in catalytic stability [21]. Hydrogenation experiments of deposited carbon species were performed using H_2_-TPH (Figure 4). The resulting profile exhibited multiple discernible peaks, thereby indicating the presence of diverse carbon compounds. The peak observed near 150 °C is attributed to the hydrogenation and removal of strongly adsorbed heavy hydrocarbons. In contrast, the high-temperature peak corresponds to the hydrogenation of more refractory carbonaceous deposits [22]. Comparative analysis indicates significant deposition of carbonaceous species on the CuZn catalyst, which is effectively converted during subsequent hydrogenation. Simultaneously, the introduction of liquid metal Ga led to a sharp decrease in the corresponding peak area, indicating a pronounced suppression of carbon deposition within the catalyst facilitated by liquid gallium. Among all tested catalysts, CuZnGa-p demonstrated the lowest amount of carbon deposition.

### 2.3. Catalytic Performance

**Figure 5:**
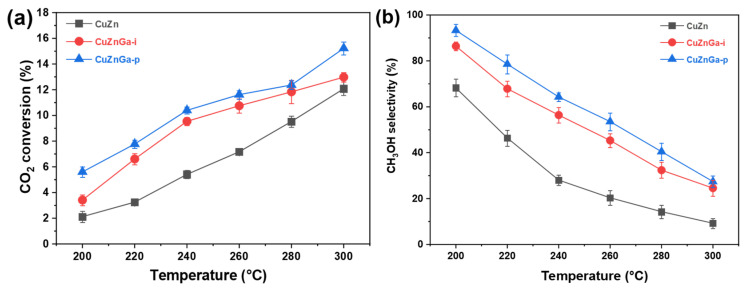
(**a**) CO_2_ conversion and (**b**) methanol selectivity of CuZn, CuZnGa-i, and CuZnGa-p catalysts under the same conditions. These catalysts were tested at 4 MPa, H_2_/CO_2_ = 3:1, and 7500 mL∙g_cat_^−1^∙h^−1^.

In the CO_2_ hydrogenation process for methanol synthesis, a significant competing side reaction is the reverse water–gas shift (RWGS) reaction (CO_2_ + H_2_ → CO + H_2_O), which leads to parallel reaction pathways and reduced selectivity toward methanol. Consequently, CO was identified as the primary by-product during product analysis, with other by-products being virtually absent. This indicates that the reverse water–gas shift reaction is the dominant side reaction, with methanol selectivity being the primary optimization challenge. The CO_2_ hydrogenation performance of the three synthesized catalysts was evaluated, as presented in Figure 5a,b. Consistent with thermodynamic expectations, elevated reaction temperatures enhanced CO_2_ conversion. However, they also favored the reverse water–gas shift (RWGS) reaction, resulting in increased CO selectivity. Consequently, higher reaction temperatures led to a reduction in methanol selectivity. As shown in the CO_2_ conversion and methanol selectivity profiles, the CuZnGa-p catalyst exhibits the highest CO_2_ conversion and methanol selectivity among the series under identical reaction conditions, followed by CuZnGa-i. In contrast, the CuZn catalyst showed the lowest activity and preferentially catalyzed the RWGS reaction, leading predominantly to CO formation. The CO_2_ conversion rate of the unmodified CuZn catalyst increased from 2.1% to 12.0% with increasing temperature, whereas the methanol selectivity decreased from 68.4% to 9.5%. In contrast, the CO_2_ conversion rate of the CuZnGa-p catalyst increased from 5.4% to 15.0% as the temperature increased, whereas the corresponding methanol selectivity declined from 94.6% to 27.3%. Considering that liquid Ga and the Cu–ZnO catalyst were only physically mixed and thus had little impact on the textural structure, the observed enhancement in CO_2_ conversion and methanol selectivity can be attributed to the high fluidity of the liquid phase, which effectively suppresses carbon deposition. This inhibition of coke formation prevents the blockage of active sites, thereby improving the overall catalytic performance.

**Figure 6:**
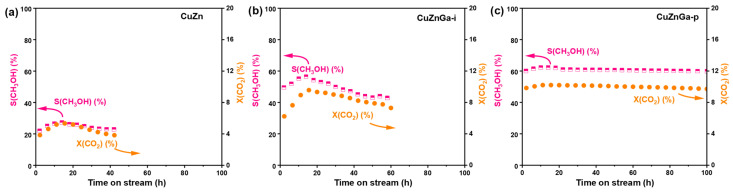
Catalysts stability test for (**a**) CuZn, (**b**) CuZnGa-i, and (**c**) CuZnGa-p at 4 MPa, H_2_/CO_2_ = 3:1, 240 °C, and 7500 mL∙g_cat_^−1^∙h^−1^.

Catalyst stability represents a critical performance metric for evaluating its practical applicability. Under identical testing conditions, the stability of the three catalysts was evaluated (Figure 6a–c). Significant disparities in catalytic lifetime were observed among them, indicating that the presence of liquid metal Ga plays a decisive role in determining catalyst durability. The pure CuZn catalyst exhibited a gradual deactivation after 40 h of stable operation. In comparison, the impregnation-synthesized CuZnGa-i catalyst demonstrated improved stability, maintaining activity for approximately 60 h. The CuZnGa-p catalyst, prepared via physical mixing, demonstrated an extended operational lifetime of up to 100 h, with only marginal deactivation observed after 80 h on stream. Its optimal catalytic performance was achieved approximately 5 h into the reaction, likely attributable to an induction period associated with the CO_2_ hydrogenation to methanol process. Comparative stability evaluation of the three catalysts reveals that the incorporation of liquid metal Ga significantly enhances resistance to carbon deposition, as proved by the H_2_-TPH characterization, thereby contributing to superior long-term catalytic stability. The improved stability of liquid metals was also proved in CO hydrogenation and methanol to hydrocarbons reactions, and the improved stability was attributed to the decreased carbon deposit because of the high mobility and surface self-cleaning of liquid metals [22,23]. Furthermore, the composition of the used CuZnGa-p catalyst shows almost no change compared with the fresh CuZnGa-p catalyst, further demonstrating the stable structure and explaining its high catalytic stability.

## 3. Discussion

The introduction of liquid metal Ga did not significantly alter the catalyst’s structure or morphology. The enhanced catalytic activity is primarily attributed to the fluidity of liquid gallium, which effectively suppresses carbon deposition. In addition, CO_2_-TPD analysis reveals that the incorporation of liquid metal Ga increases the density of alkaline sites, thereby facilitating CO_2_ activation and reducing the RWGS reaction. H_2_-TPR analysis illustrates that the liquid metal Ga has a strong interaction with Cu-ZnO catalysts and reduces its reduction temperature. The formation of carbon deposits was quantified by H_2_-TPH, and the long-term stability was assessed for all three catalysts. In terms of catalytic performance, the introduction of liquid metal Ga significantly enhances both the CO_2_ conversion rate and methanol selectivity relative to the impregnated CuZnGa-i and unmodified CuZn catalysts. Although Ga is liquid at 300 °C, its distribution is route-dependent: CuZnGa-p retains discrete, perimeter-pinned GaOx domains that expand the Cu–(GaOx)–ZnO triple interface and oxygen-vacancy density, whereas CuZnGa-i forms more continuous GaOx coverage that partially masks Cu–ZnO contact; this interfacial contrast, not the formation of new bulk phases, accounts for the higher CO_2_ activation and methanol selectivity of CuZnGa-p. Comparative studies with other catalysts further confirm that CuZnGa-p achieves superior activity and selectivity (Table 2), demonstrating competitive CO_2_ conversion and CH_3_OH selectivity relative to state-of-the-art systems. Therefore, based on comprehensive performance evaluation, phase structure characterization, chemical behavior analysis, and stability assessments, the introduction of liquid gallium effectively suppresses carbon deposition and consequently enhances the hydrogenation of CO_2_ to methanol.

## 4. Materials and Methods

### 4.1. Material Preparation

All chemicals were used as received without further purification. Copper(II) nitrate trihydrate (Cu(NO_3_)_2_·3H_2_O, 99.9%) was supplied by Thermo Fisher Scientific Inc. (Waltham, MA, USA). Zinc nitrate hexahydrate (Zn(NO_3_)_2_·6H_2_O, 99.9%) was obtained from Alfa Aesar (China) Chemical Science Co., Ltd. (Shanghai, China). Gallium (Ga, 99.99%) was provided by Beijing Innochem Technology Co., Ltd. (Beijing, China). Liquid gallium typically solidifies at temperatures above 30 °C. Sodium carbonate (Na_2_CO_3_, analytical grade) was sourced from Sinopharm Chemical Reagent Co., Ltd. (Beijing, China). Deionized water was produced in-house and used throughout the experiments.

### 4.2. Catalyst Preparation

CuZn catalyst: Dissolve Cu(NO_3_)_2_·3H_2_O and Zn(NO_3_)_2_·6H_2_O in a molar ratio of 6:4 in 150 mL of deionized water, and prepare a 0.1 mol/L sodium carbonate solution to be used as the precipitating agent. Under continuous stirring conditions, add both solutions dropwise into a 1000 mL beaker containing 100 mL of deionized water maintained at 70 °C in a water bath. Upon complete precipitation, the resulting suspension was aged at the same temperature for 2 h. The precipitate was subsequently collected by filtration and washed repeatedly with deionized water to remove residual Na ions. The solid was then dried overnight at 110 °C. The obtained precursor was calcined under a flowing air atmosphere at 550 °C for 5 h to yield the final product, which is denoted as CuZn.

CuZnGa (impregnation method) catalyst: Using the previously synthesized CuZn catalyst as a starting material, 1 g of the catalyst was dispersed in 150 mL of deionized water. Then, 0.05 g of liquid gallium was added to the suspension. The mixture was stirred in a water bath maintained at 70 °C until complete dryness was achieved. The resulting solid was subsequently calcined under a flowing air atmosphere at 550 °C for 5 h to obtain the final catalyst, designated as CuZnGa-i.

CuZnGa (physical mixing method) catalyst: Liquid gallium was first solidified and ground into a fine powder using a scraper. The Ga powder and CuZn were then combined and thoroughly shaken in a polypropylene centrifuge tube at room temperature (20–25 °C), ensuring the temperature remained below Ga’s melting point so that Ga stayed solid throughout mixing. Specifically, 0.05 g of the resulting Ga powder and 1.0 g of the previously synthesized CuZn catalyst were weighed and combined in a centrifuge tube. The mixture was thoroughly shaken to achieve a homogeneous physical blend, yielding a catalyst denoted as CuZnGa-p.

### 4.3. Characterization

The crystal structure of various catalysts was tested by X-ray diffraction (XRD, Bruker D8 Advance, Staufen, Germany). Scanning transmission electron microscopy (STEM) and energy-dispersive X-ray spectroscopy (EDS) analyses were carried out on a JEOL JEM-F200 field-emission electron microscope (JEOL, Akishima, Japan) with an acceleration voltage of 200 kV. Inductively coupled plasma mass spectrometry (ICP-MS) was performed on an Agilent 7500a apparatus (Santa Clara, CA, USA). Before analysis, the samples were dissolved using aqua regia and diluted. Temperature-programmed reduction of H_2_ (H_2_-TPR), temperature-programmed desorption of CO_2_ (CO_2_-TPD), and temperature-programmed hydrogenation of H_2_ (H_2_-TPH) were conducted on a Micromeritics Autochem II chemisorption analyzer (Shanghai, China). During TPR, the reduction profile of the catalyst was recorded from 50 to 500 °C in a 5% H_2_/Ar (in volume, 30 mL min^−1^) mixed gas. For TPD analysis, the catalyst was first pretreated at 400 °C under a 5% H_2_/Ar flow for 60 min and then cooled to 50 °C. Subsequently, the gas was switched to argon for purging over a period of 30 min. This was followed by adsorption under a 10% CO_2_/Ar mixture for 60 min. The system was then flushed again with argon for 60 min to remove any physisorbed or gas-phase hydrogen. Finally, after the baseline had stabilized, the desorption profile was recorded from 50 to 800 °C under argon flow. For TPH, the post-reaction catalyst was heated from room temperature to 800 °C under a 5% H_2_/Ar flow at a rate of 30 mL min^−1^. The heating rate for all chemical adsorption experiments is 10 °C min^−1^.

### 4.4. Catalytic Activity Test

The catalytic performance of all synthesized catalysts was evaluated using a high-pressure fixed-bed reactor. In a typical procedure, 0.2–0.3 g of catalyst was loaded into an 8 mm inner diameter reaction tube. An in situ reduction treatment was first conducted under a 10% H_2_/Ar flow at 400 °C for 5 h. After cooling to room temperature under the same atmosphere, the reactor was pressurized to the target value, and the temperature was programmed to rise under a flow of H_2_/CO_2_ reaction gas to initiate the catalytic reaction. The flow rates of all gases were regulated by mass flow controllers, and the gas lines were maintained at 150 °C to prevent condensation of reaction products. The effluent gases were analyzed using an online gas chromatography system (GC 2060) equipped with both a thermal conductivity detector (TCD) and a flame ionization detector (FID). The TCD, using helium as the carrier gas, was employed for the analysis of Ar, CO, and CO_2_. The FID, with nitrogen as the carrier gas and 8% Ar as an internal standard, was used to quantify alcohols and other carbon-containing compounds. All experimental data were collected after 2 h of reaction to ensure steady-state operation, and each catalyst was tested three times to verify the reproducibility of the results. Furthermore, the same experimental results were obtained through repeated experiments using three different batches of samples.

## 5. Conclusions

In this study, CuZn catalysts were modified with liquid metal Ga using different introduction methods to evaluate their impact on catalytic performance and stability for CO_2_ hydrogenation to methanol. Activity tests indicated that the CuZnGa-p catalyst, prepared by physical mixing, achieved the highest CO_2_ conversion, methanol selectivity, and long-term stability. Characterization results further demonstrated that the CuZnGa-p catalyst exhibits an increased number of basic sites, which facilitate CO_2_ activation, while effectively suppressing carbon deposition, thereby ensuring long-term stability. This work offers theoretical insights into the application of liquid metals in CO_2_ hydrogenation catalysis and proposes a novel strategy for the design of efficient and stable catalysts for methanol synthesis from CO_2_.

## Figures and Tables

**Table 1:** The elemental analysis of the catalysts by ICP-MS.

Samples	Percent of Cu (%)	Percent of Zn (%)	Percent of Ga (%)
CuZn	78.9	21.1	-
CuZnGa-i	74.1	17.6	8.3
CuZnGa-p	75.9	15.2	8.9
CuZnGa-p (used)	76.1	15.1	8.8

**Table 2:** Comparison of CO_2_ conversion and methanol selectivity of catalysts with this work.

Catalyst	CO_2_ Conversion (%)	Methanol Selectivity	Temperature (°C)	Pressure (MPa)	Refenence
CuZnGa-p	10.2	64.4	240	4	This work
Cu(ZnGa)	3.4	35.4	250	3	[24]
GaZrO_x_	2.30	73.0	300	2	[25]
CuGa_10_/SiO_2_	1.9	11.0	280	0.8	[26]
Cu_10_Ga_10_/SBA-15	<0.5	100	220	0.5	[27]

## Data Availability

The data supporting this study are available from the corresponding author upon reasonable request but are not publicly available due to privacy restrictions.
